# Looking behind the score: Skill structure explains sex differences in skilled video game performance

**DOI:** 10.1371/journal.pone.0197311

**Published:** 2018-05-30

**Authors:** Kyle W. Harwell, Walter R. Boot, K. Anders Ericsson

**Affiliations:** Department of Psychology, Florida State University, Tallahassee, Florida, United States of America; Ludwig-Maximilians-Universitat Munchen, GERMANY

## Abstract

Some have explained large sex differences in visuospatial abilities by genetic adaptations to different roles in primitive hunter-gatherer societies and the interaction of innate biological differences and environmental factors. We explored the extent to which variations in behavior and acquired skills can provide alternative accounts for sex differences in the performance of a complex spatially-demanding video game (Space Fortress). Men and women with limited video game experience were given 30 hours of training, and latent curve analyses examined the development of their ship control performance and behavior. Men had significantly better control performance than women before and after training, but differences diminished substantially over the training period. An analysis of participants’ joystick behaviors revealed that initially men and women relied on different patterns of control behaviors, but changes in these behaviors over time accounted for the reduced sex differences in performance. When we controlled for these differences in behavior, sex effects after training were no longer significant. Finally, examining the development of control performance and control behaviors of men and women categorized as initially high and low performers revealed the lower-performing women may have been controlling their ship using an approach that was very different from the men and higher-performing women. The potential problems of analyzing men and women’s spatial performance as homogenous groups are discussed, as well as how these issues may account for sex differences in skilled video game performance and perhaps other domains involving spatial abilities.

## Introduction

### Sex differences in spatial abilities: Anthropological and biological perspectives

The origin of sex differences in spatial ability has been a topic of much academic interest over the years, and a number of studies have reported men demonstrating superior performance compared to women on spatial tasks like mental rotation [1,2]. Men have also demonstrated advantages in tasks like spatial navigation, possibly due to employing more efficient, biologically predisposed strategies for wayfinding [3,4]. Some researchers have proposed an evolutionary explanation for this advantage, notably the hunter-gatherer hypothesis of spatial sex differences, which suggests superior visuospatial abilities played a crucial role in the tracking and killing of elusive and quick-moving prey and provided a selective advantage in male humans for many thousands of years [5,6]. According to this view there are innate, biological mechanisms developed during evolutionary history that explain why men outperform women on a variety of visuospatial tasks. Other lines of research have also expanded on the anthropological hypotheses by providing additional biological evidence for differences between men and women’s spatial cognition. Recent investigations have found that women who were exposed to higher than normal levels of prenatal androgens displayed better spatial ability performance compared to women who were not, suggesting a link between the development of spatial abilities and the presence of male sex hormones [7,8]. In addition to potential biological sex differences in spatial abilities, societal and environmental factors likely play an important role in explaining the male advantage in performance of spatially-demanding tasks. For example, the biopsychosocial model proposed by [9,10] describes a positive feedback cycle in which biological dispositions and differences in brain structure and organization bias how individuals select their environments, and environmental factors then reciprocally influence biological development. The interaction with environmental elements becomes especially important when considering sex differences in light of differences in the amount of engagement with activities believed to be associated with spatial skill development.

### Sex differences in spatial abilities: Sociocultural and strategic perspectives

Contrasting with the innate sex differences view, other researchers have proposed that the magnitude of performance differences between men and women may depend on a number of environmental factors and the context in which the studies take place, citing a number of studies failing to find any significant differences [11]. One compelling argument posits that sex differences in performance of tasks like mental rotation and spatial navigation may be partly attributable to cultural biases and gender norms that encourage boys and girls to engage in stereotypically “appropriate” behavior defined by their culture, resulting in girls getting less opportunity to participate in activities such as sports or video game play that promote development of the relevant skills [12–14]. It is also possible that women may not find these types of tasks as engaging as men do [14], or perhaps feel less confident in their ability to perform the task [15]. A further motivational factor that likely affects performance is the possibility that women may hold gender beliefs that men are typically better at spatially-demanding tasks, and this stereotype threat may negatively impact their performance and inflate measured sex differences [16,17]. A final consideration is that observed sex differences in visuospatial tasks may not reflect deficits in spatial abilities and rather reflect difficulties converting cognitive processes into motoric responses. Most studies measure spatial cognition by the speed and accuracy of button presses, and if women have less experience with tasks like video games that require many specifically-timed button presses, the response modality itself may bias measures of performance in favor of the men in the study [18]. The motoric component of sex differences in mental rotation has been studied by neuroimaging data, where findings show differential activation in regions of motor cortex that suggests sex differences in strategy [19].

The relations between sociocultural factors and performance on spatially-demanding tasks are necessarily correlational in nature, making it difficult to infer any causal mechanisms. To gain information about the mechanisms, researchers have started to investigate the behavior men and women engage in during the tasks themselves. If men and women were found to employ different strategies while performing tasks that measure spatial abilities, then at least some of the male performance advantage may be accounted for by women’s selection of different strategies and patterns of behavior. Studies have found that women were more likely than men to use a less efficient piecewise or analytic strategy rather than a holistic or rotational strategy on mental rotation tasks [20–22], and that sex differences in performance can be significantly reduced through training and manipulations of the instructions or task [17,23]. Similar differences in selected strategies have been found in examinations of men and women’s navigation of virtual environments [24,25]. This pattern of results have led some researchers to suggest the differences in selected strategies may arise from women’s tendency to process spatial information locally, whereas men tend to process spatial features globally or holistically [22,26]; thus, differences in how men and women allocate spatial attention while performing tasks may bias their selection among available strategies.

While studies such as these have provided important insights into potential strategy differences between men and women, many are limited by their assessment of participants’ strategies. For example, strategy use has been mostly inferred indirectly through analyses of overall task performance measures like accuracy and reaction time, aggregated across entire groups [20–23]. Other studies have relied on post-hoc measures of strategy like behavior on a critical trial in the task [24] or post-task self-reports of strategy use [25], and these two approaches are insensitive to changes in behavior participants may employ over the course of many trials. These strategy evaluations also fail to relate task performance to specific behaviors, making it difficult to determine what behavioral differences distinguish high and low performance within and across groups using different strategies. Research on men and women’s developmental trajectories, where strategy use may change over time as task-specific knowledge and skills are gained, requires a different approach. Such an assessment of strategies should analyze participants’ detailed behavior on individual trials and explicate how differences in behavior influences task performance. In sum, a description of men and women’s behavior while performing spatial tasks will be critical for understanding the source of observed sex differences and inform the design of more effective training procedures.

### Training spatial abilities with video games

Video games have become an increasingly important paradigm for studying complex skills, including those involving visuospatial abilities, and they offer a rich context within which to study sex differences. One area of interest concerns identifying the source of large sex differences seen across various game genres, where men demonstrate an advantage in performance for action video games like first-person shooters [27]. This has led some researchers to propose a causal relationship between lower spatial abilities and lower performance of women in spatially-demanding tasks like video games [25,28–31]. Action video games, which require players to monitor multiple features and/or track multiple targets while making time-sensitive decisions in highly dynamic environments, have been proposed as a domain well suited for studying development of generalizable spatial abilities in both non-gamers, generally [32,33], and women, specifically [30,34].

Many studies examining differences in visuospatial ability and the effects of training to reduce them [35], including those involving games, make the unverified assumption that men and women are performing the tasks using the same or similar sequences of cognitive processes. Thus, men are assumed to outperform women due to their greater capacities for spatial abilities. However, it is also possible men and women approach tasks using fundamentally different patterns of behavior. According to the expert-performance framework [36,37], there is evidence skilled performers employ qualitatively different approaches compared to unskilled performers, due to qualitatively different representations of the demands of the tasks. The lower performance seen among women may be in part attributable to less efficient patterns of behavior, which can only be identified by recording and analyzing detailed sequences of behaviors executed during performance of the tasks. Unfortunately, many studies analyze only summary scores of overall performance or focus on simple behavioral measures like reaction time which can obscure meaningful differences in participant behavior. Further, most video game studies rely on data summarized across entire gaming sessions, likely because they utilize commercial video games designed primarily for entertainment which make it difficult or impossible to extract detailed behavioral data [38]. Without the analysis of detailed sequences of behavior, the strategies mediating skilled performance may go unnoticed [39], and the skill mechanisms underlying sex differences will remain elusive.

### The present study

One skill that may be especially important to consider when evaluating player performance is the ability to manipulate the player’s avatar within the game. Given the complex demands action video games impose on players, players would not be expected to achieve a high overall score if they lack the prerequisite skills necessary to maneuver around the game space. Thus, measurements of how well players are controlling their avatars and what behaviors discriminate good versus bad control would likely account for individual differences in development of skilled action video game performance in these types of games. Additionally, examination of control-relevant behaviors could provide important details regarding the intermediate stages that participants progress through as they develop or alter their control strategies as a result of training.

The current investigation was motivated by the possibility of extending beyond the existing sex differences literature by examining how online measures of participant behavior may provide insight into the relations between sex and differences in assessed strategies and task performance. We analyzed sex differences in development of control performance in an action video game (Space Fortress; [40]) and how participants’ control behaviors changed over the course of training. We also evaluated whether the sex effects before and after training persisted after statistically controlling for players’ control behaviors. Finally, we compared development of control performance and control behaviors among participants within each sex for individuals identified as low and high performers based on their performance before training.

## Methods

### Participants

We analyzed an archival dataset collected by a research group at the University of Illinois at Urbana-Champaign [41] to investigate the effect of two training regimens on development of Space Fortress performance. For the present investigation, we excluded the 25 participants in the no-training control group that lacked comparable data on training to model growth. Given that the two training conditions did not differ in their overall performance (*F*(1, 46) = 3.78, *p* = .058, η^*2*^ = .076) or their Control score performance (*F*(1, 46) = 1.23, *p* > .1, η^*2*^ = .026), participants in both groups were pooled into a single sample for the current analyses. We studied the performance of 50 young adults (aged 18–30, 31 women). All participants were paid for completing approximately 60 hours of testing and training across multiple sessions. Participants had been pre-screened to ensure that none had extensive video game playing experience (defined as more than 4 hours of gameplay per week). All reported normal or corrected-to-normal vision, normal color vision, and right-handedness. The Internal Review Board of the University of Illinois approved original data collection, and all participants provided written informed consent according to the principles of the Declaration of Helsinki. The present investigation involved no intervention or interaction with participants, and no personally identifiable or private data were used in any analyses; as such, the Florida State University Human Subjects Committee did not consider this research to require additional review.

### The Space Fortress video game

Space Fortress (SF) is a video game originally designed by cognitive psychologists that combines complex motor movements, working memory for targets, multi-tasking demands, simultaneous resource monitoring, and considerable spatial attention requirements [40,41]. The player’s primary objective is to maximize their total score by firing missiles to destroy the enemy fortress (located in the center of the game environment) as many times as possible over the course of a 3-minute game. A player’s total score is the sum of their performance across several subdomains of gameplay, each with its own accompanying subscore.

**Fig 1:**
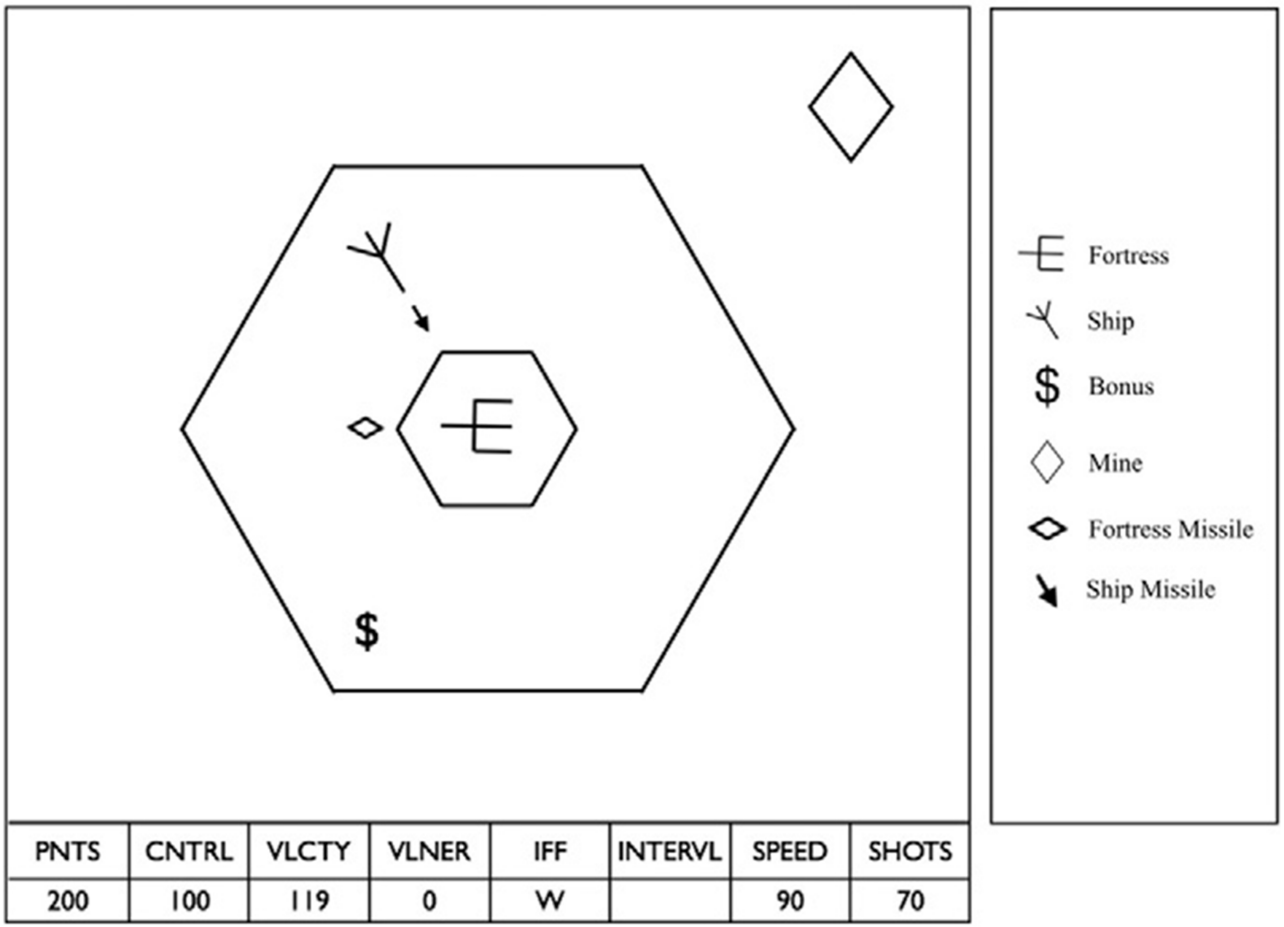
Space Fortress game environment. Large hexagon indicates boundary for maximal Control score zone. Small hexagon indicates boundary for a fortress collision.

The Control score is based on how well players maneuver their ship within the two-dimensional, frictionless game environment. Using the joystick they are able to rotate their ship clockwise or counterclockwise and accelerate the ship forward along its current angle of orientation by pushing the joystick forward (executing a thrust). The game samples the player’ ship position twenty times every second, and every twenty cycles it checks whether the ship is within the hexagonal boundary and updates the Control score accordingly. Players gain 7 points every time they are within the hexagonal boundary when the game updates their Control score, and they lose 7 points if they are outside the boundary. Players are also penalized for colliding with the fortress (minus 5 points), and flying beyond the edges of the game space (minus 35 points). All players start each game with a Control score of 0 and can achieve a maximum attainable Control score of 1260 (earning 7 points for all 180 Control score updates). See Fig 1 for an illustration of the SF game environment. We focus on Control score for two reasons: 1) it is the most spatial aspect of the game, making it of primary interest for studying sex differences, and 2) ship control is critical for performing all other tasks associated with SF that influence Total score. Without adequate control of the ship, destroying the fortress or efficiently dealing with mines becomes virtually impossible.

In a number of ways this study provides important extensions of previous studies of video game training, generally, and SF training, specifically (e.g., [41,42]). First, SF affords a few notable advantages over the commercial games used in other studies. SF outputs a record of game events from which we can extract player behavior on a per-game basis, in addition to calculating performance summary scores to track player development over time. Additionally, the control interface in SF is very different from modern video games and reduces the chances that participants would have significant transferrable experience using similar interfaces prior to the beginning of training. A further motivation for this study was previous SF analyses have not considered sex as a factor of primary interest, instead including it as a covariate and not reporting potentially informative sex differences in SF performance and development.

### Procedure

Participants completed 15 sessions of training with SF, each lasting about 2 hours (for full details regarding the specific training methodology, see [41]). During each session, participants played a total of thirty-six 3-minute games with the first three and last three games of each session being considered “test” trials and the remaining thirty trials being considered “training” trials. Data from the SF video game were collected by computers connected to a common network, with players executing game inputs using a computer mouse and a Logitech Attack 3 Joystick^®^. The game was presented on color 19” LCD computer monitors. For the present investigation, all analyses focus on Control scores and control behaviors averaged across the final three 3-minute test trials of each training session. Data for a single session for 3 participants were excluded due to the absence of any recorded player behavior, leaving 747 total observations for each measure.

## Statistical analyses

### Analysis 1: Effect of sex on spatial control development

To gain insight into the rate of change in Control score, latent curve analysis was used to examine the development of that score in the Space Fortress video game across 15 training sessions. All models of the data were fitted with logarithmic growth curves, with a model including only the effects of training session as the base model, and then the fixed effects of sex (women as reference group) on the intercept and linear time terms were added after. Intercepts and slopes were allowed to vary freely across participants for each model, and model fit comparisons were assessed using -2 times the change in log-likelihood. All latent curve modelling in the present investigation was performed using the lme4 package [43] in the R programming language for statistical computing [44].

### Analysis 2: Effect of sex on development of detailed control behaviors

We hypothesized the reduction in sex differences in control performance might reflect a modification of how men and women approach ship control over time and result in changes in execution of detailed control behaviors across training. Comparisons between latent curve models were used to test the effects of sex on the developmental trajectory of the three ship control behaviors (i.e., thrusts, clockwise rotations, and counterclockwise rotations) across the 15 training sessions.

### Analysis 3: Sex differences after training are accounted for by differences in control behaviors

We hypothesized the three types of detailed control behavior would account for all reliable variance in Control score. Two hierarchical regression analyses were conducted, the first predicting Control score for the first training session (where sex differences were largest), and the second focusing on Control score for the final training session (where sex differences were smallest). For both models, ship thrusts, clockwise rotations, and counterclockwise rotations were entered at the first level, and then sex was entered at the second level.

### Analysis 4: Effect of sex differs for high and low performers

Our final question examined whether sex differences in control skill development are influenced by different patterns of control behaviors executed by men and women. Specifically, we hypothesized the large sex differences seen prior to training were attributable to some women employing less efficient patterns of control behavior, and the reduction in sex differences after training was related to their adoption of behavior more consistent with the men’s behavior. Towne, Boot, and Ericsson [45] previously described participants employing vastly different patterns of behavior for controlling the SF ship (strategies) with relationships to differential game performance, and Destefano and Gray [46] pointed to skilled players’ ability to develop highly-specialized strategies to exploit particular game mechanics to improve their scores. If lower-performing players are employing qualitatively different patterns of behavior, then analyses looking at performance and behaviors aggregated across high and low performers may not be appropriate for explaining the sex effects.

Men and women were categorized as high or low initial performers based on their performance for Session 1 by a median split of their Control scores for each sex. Latent curve analyses were conducted on the development of Control score and the three ship control behaviors following the same procedures as above, this time adding an additional model that included the effect of initial performance (low performers as reference group), its interactions with sex and training session, and the three-way initial performance by sex and training session interaction.

## Results

### Analysis 1: Effect of sex on spatial control development

**Fig 2:**
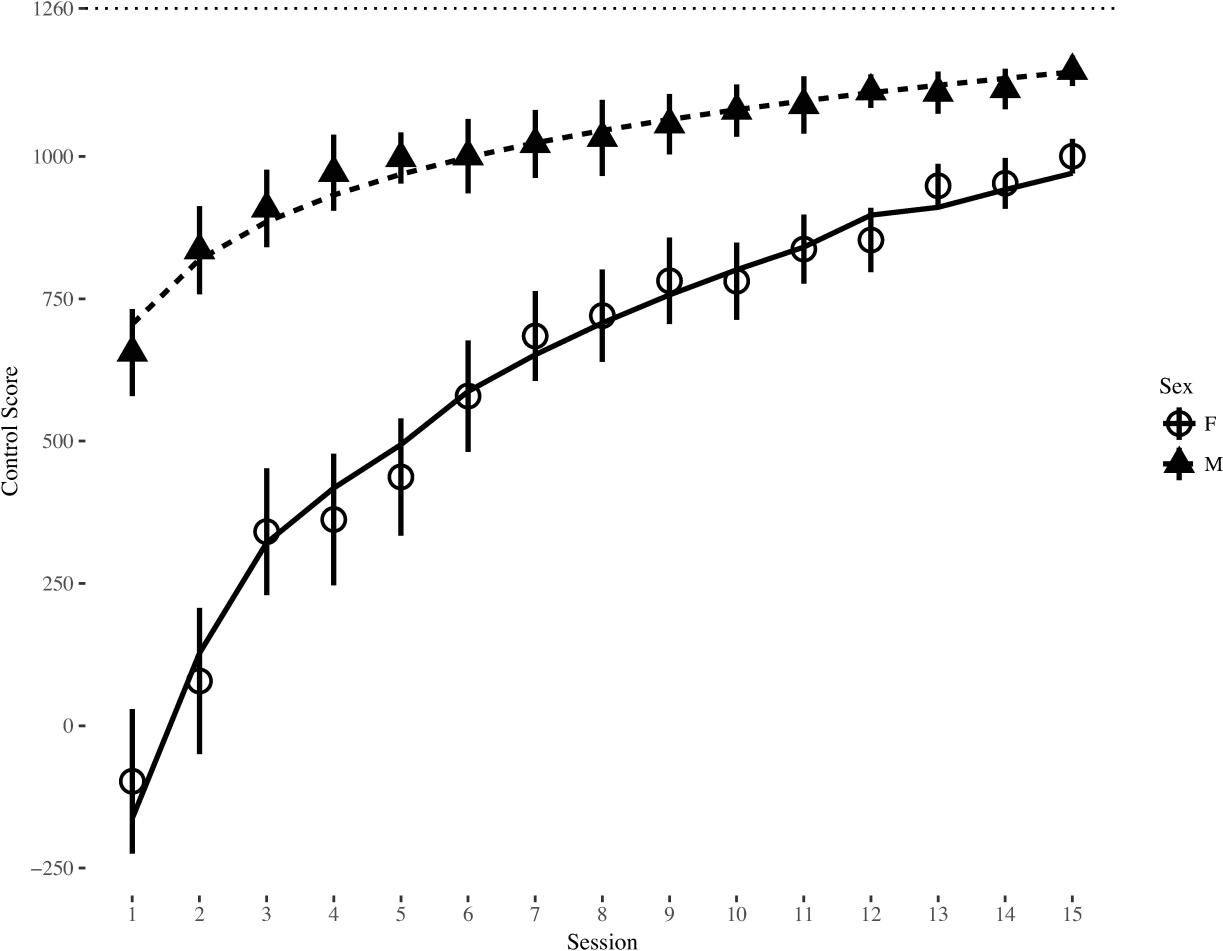
Control scores by sex across training session. Error bars represent standard error of the mean. Dotted line represents maximum achievable Control score.

The effect of sex significantly improved model fit (Δχ^2^(2) = 17.16, *p* < .001), and examination of parameter estimates of the final model revealed a significant effect of session (*b* = 3.43, *t*(49.94) = 11.03, *p* < .001), indicating a significant improvement in Control score across training sessions. There was also a significant effect of sex on the intercept (*b* = 868.17, *t*(49.96) = 4.52, *p* < .001), indicating that men started training with significantly higher Control performance (Session 1 Cohen’s *d*_*sex*_ = -1.50). In addition, the interaction between sex and session was significant for the slope (b = -586.94, *t*(49.92) = -4.14, *p* < .001), suggesting women improved significantly faster than men and the magnitude of the sex difference significantly decreased over time. Rerunning the models with the intercept set at the final training session revealed the effect of sex was still significant but substantially smaller (*b* = 177.88, *t*(49.92) = 2.49, *p* = .016, *d*_*sex*_ = -0.95). See Fig 2 for a graphical depiction of Control score over time.

### Analysis 2: Effect of sex on development of detailed control behaviors

**Fig 3:**
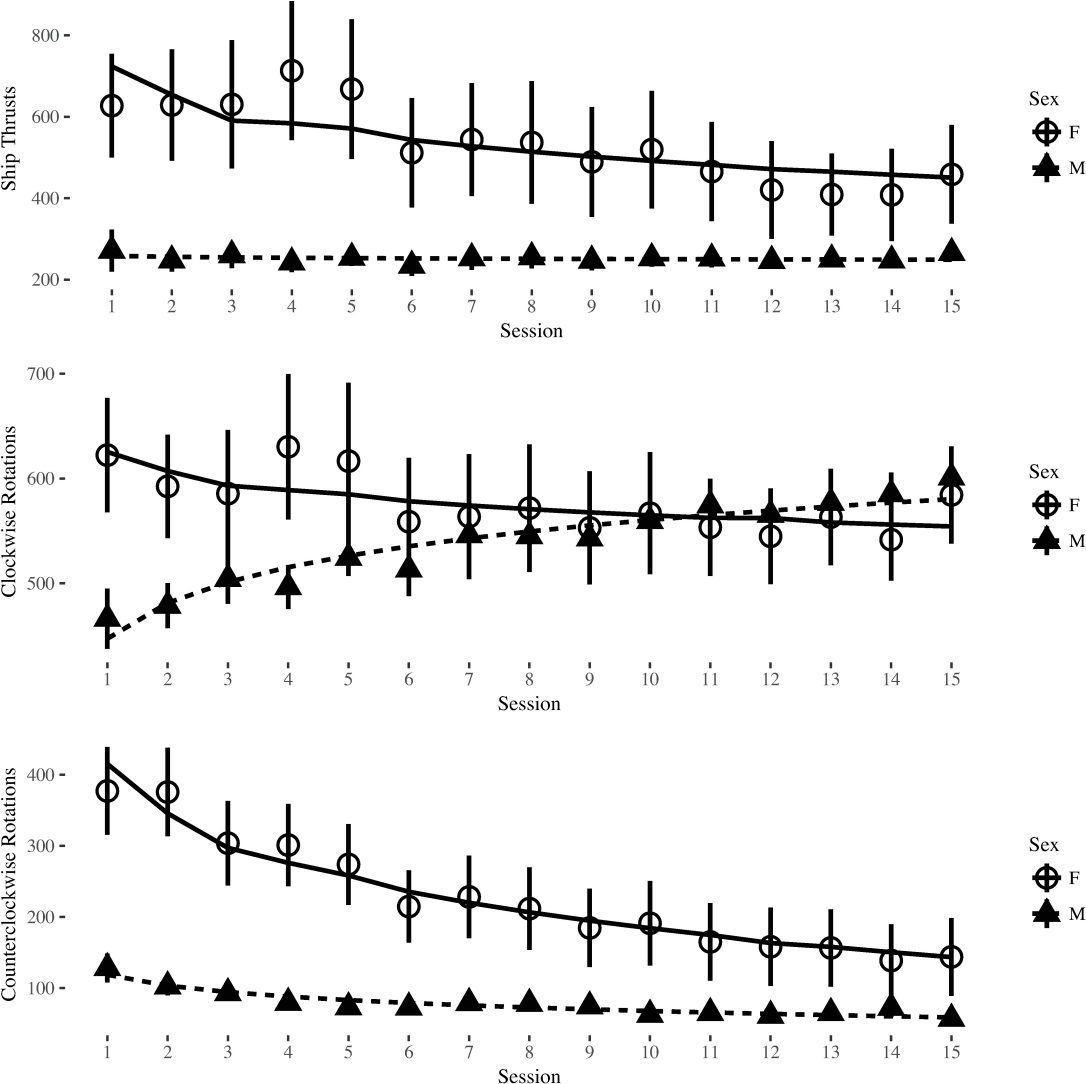
Player ship control behaviors by sex across training session. Error bars represent standard error of the mean.

The models including the effects of sex displayed significant improvement of fit beyond the base models for clockwise rotations, Δχ^2^(2) = 7.16, *p* = .028, and counterclockwise rotations, Δχ^2^(2) = 15.81, *p* < .001. Sex did not significantly improve fit beyond the base model for ship thrusts, but the difference approached significance, Δχ^2^(2) = 4.94, *p* = .085. There was a significant sex by training session interaction for the developmental trajectory of both types of ship rotations (clockwise rotations, *b* = 173.74, *t*(49.90) = 2.76, *p* = .008; counterclockwise rotations, *b* = 180.14, *t*(49.98) = 2.39, *p* = .021). Women began their training executing significantly more rotations per game than men (clockwise rotations, b = -178.24, *t*(49.99) = -2.07, *p* = .044, *d*_*sex*_ = 0.62; counterclockwise rotations, b = -296.62, *t*(49.99) = -3.31, *p* = .002, *d*_*sex*_ = 0.90), but as training progressed they reduced their numbers of ship thrusts and rotations significantly more than men did. When re-analyzed using the intercepts set at the final training session there were no significant differences in behaviors executed by men and women at the end of training (clockwise rotations, *b* = 26.09, *t*(49.90) = 0.41, *p* > .250, *d*_*sex*_ = -0.08; counterclockwise rotations, *b* = -1.40, *t*(49.80) = -0.11, *p* > .250, *d*_*sex*_ = 0.36; see Fig 3 for graphs of thrusts, clockwise rotations, and counterclockwise rotations across training session). These analyses show that sex differences in Control performance can be associated with differences between men and women’s Control behaviors.

### Analysis 3: Sex differences after training are accounted for by differences in control behaviors

For the analysis of the first session (Session 1), the final model explained a significant portion of the variance in Control scores (*R*^*2*^ = .62, *F*(4, 45) = 18.56, *p* < .001), and sex contributed significantly above and beyond the Control behaviors, Δ*R*^*2*^ = .06, *F*(1,45) = 7.69, *p* = .008. For this session the parameter estimates for thrusts and counterclockwise rotations significantly predicted Control scores (thrusts: *b* = 0.64, *t*(45) = 3.04, *p* = .004; counterclockwise rotations: *b* = -2.36, *t*(-5.59), *p* < .001). At the final session (Session 15), the final model also explained a significant portion of the variance in Control scores (*R*^*2*^ = .67, *F*(4, 45) = 22.36, *p* < .001). However, sex did not explain variance in Control score above and beyond the three behaviors after training (Δ*R*^*2*^ = < .001, *F*(1,45) = 0.03, *p* > .250). Parameter estimates revealed all three Control behaviors were predictive of performance (thrusts: *b* = -1.23, *t*(45) = -7.56, *p* < .001; clockwise rotations: *b* = 1.61, *t*(45) = 6.42, *p* < .001; counterclockwise rotations: *b* = 1.23, *t*(45) = 5.45, *p* < .001).

### Analysis 4: Effect of sex differs for high and low performers

For all outcome measures, the addition of the models with initial performance significantly improved fit compared to the models that only included the effects of sex (Control: Δχ^2^(4) = 47.76, *p* < .001; thrusts: Δχ^2^(4) = 12.02, *p* = .017; clockwise rotations: Δχ^2^(4) = 9.85, *p* = .043; counterclockwise rotations: Δχ^2^(4) = 26.68, *p* < .001).

**Fig 4:**
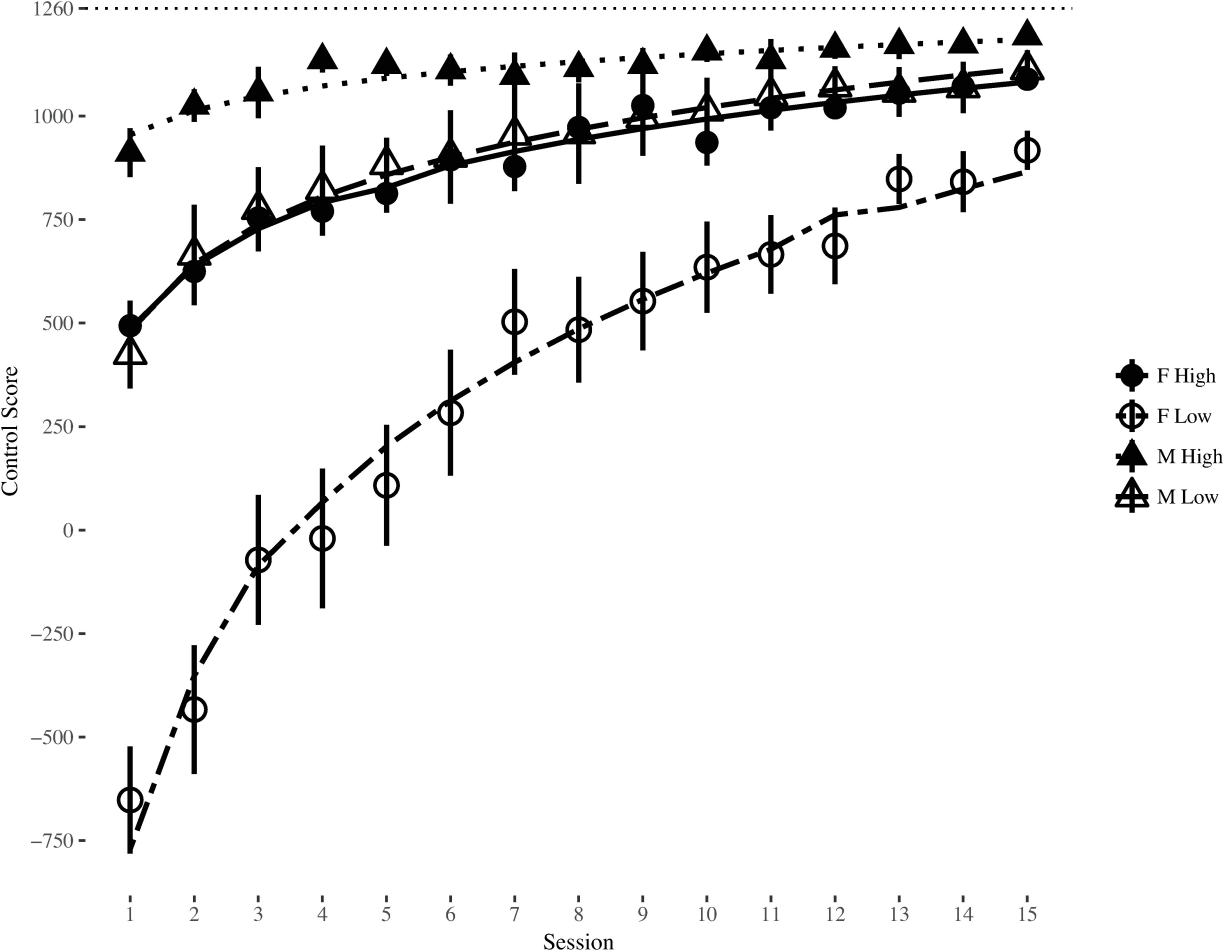
Control scores by sex and initial control performance across training session. Error bars represent standard error of the mean. Dotted line represents maximum achievable Control score.

**Fig 5:**
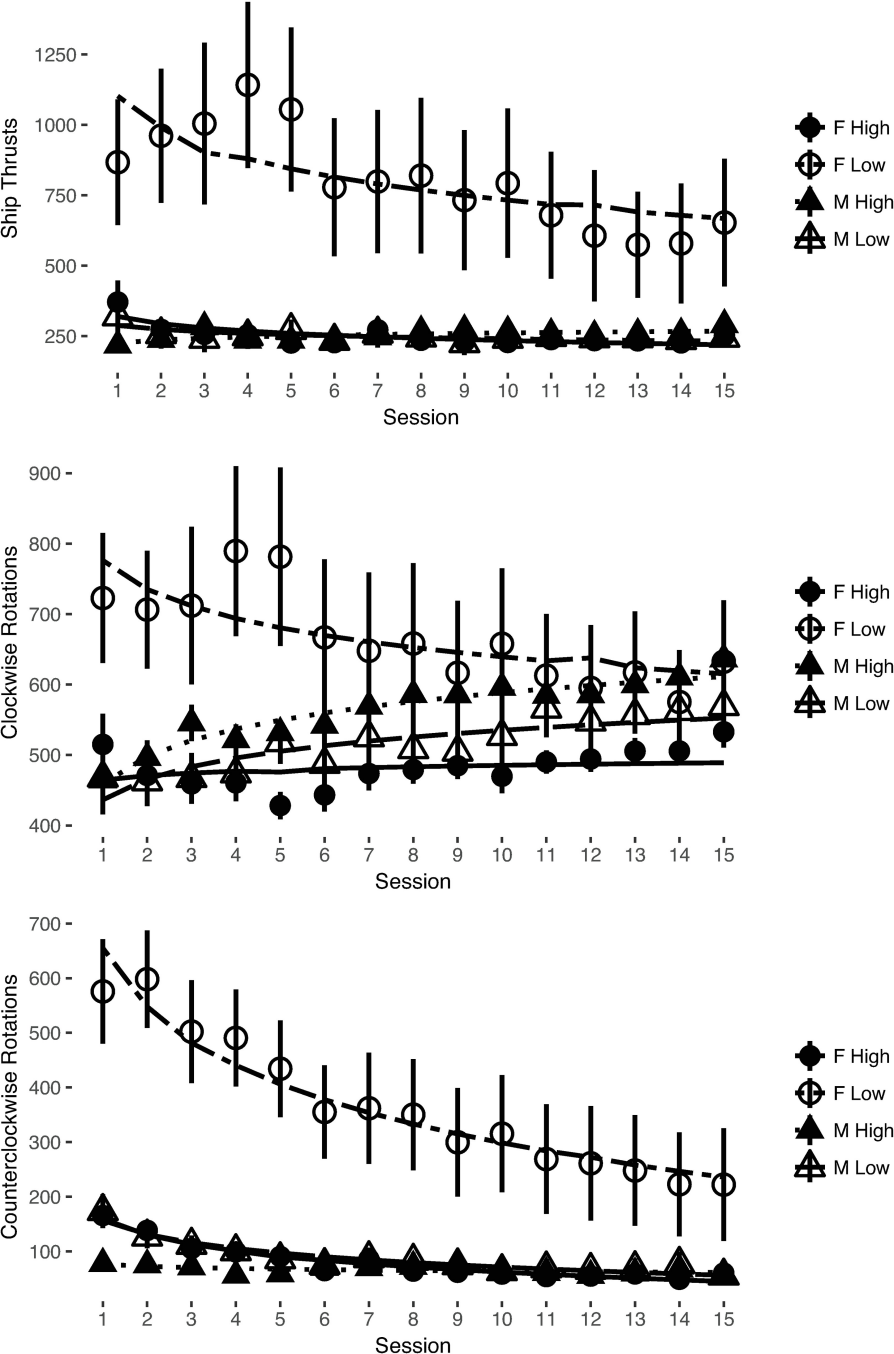
Player ship control behaviors by sex and initial control performance across training session. Error bars represent standard error of the mean.

**Fig 6:**
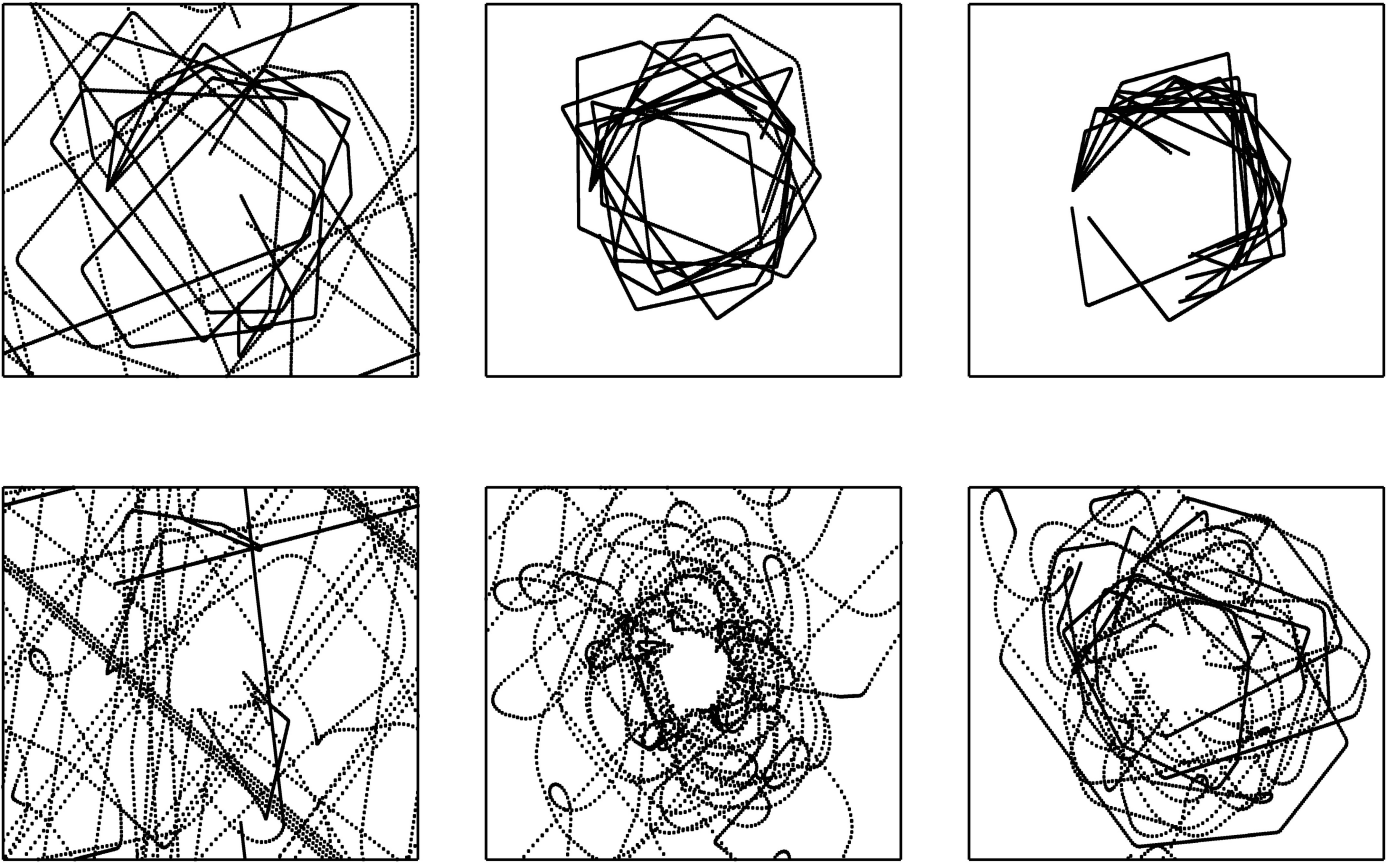
Example flightpaths of high and low-performing women across training. *Top Row*, *from Left*: Flightpaths for final games of Session 1, 8, and 15 for the highest-performing female participant. *Bottom Row*, *from Left*. Flightpaths for final games of Sessions 1, 8, and 15 for the lowest-performing female participant. Dotted lines indicate ship position over time, and borders indicate edges of the game space. Note increased spacing between points for the low-performing player, indicating greater ship velocity.

Examination of parameter estimates (see Table 1) of the final models revealed a significant effect of initial performance, as well as sex by initial performance and training session by initial performance interactions for Control score and both ship rotations. Additionally, the three-way interaction was also significant for Control score. Taken together, these findings suggest the large sex effects on control performance observed prior to training were different for initially high and low performing men and women, and these differences were also associated with similar differences in ship control behaviors. Moreover, the previously described sex differences in development of Control score across training was dependent upon whether players were high or low performers at the beginning of training (see Fig 4). Initially low performing women executed more of all control behaviors than high performing women and both high and low performing men prior to training, and they steadily reduced their number of actions over time (see Fig 5). This suggested some women were employing significantly different ship control behavior early on and modifying their gameplay over time. Indeed, visual inspection of the flight paths of players seemed to suggest low-performing women with high numbers of thrusts had a less systematic pattern of flight compared to more skilled women, but they became more consistent across training (see Fig 6 for example flight paths).

**Table 1:** Parameter estimates for final models.

* *	Coefficient	Std. Error	t-Statistic	Prob.
Control				
Sex	1251.86	165.48	7.57	< .001
Session	1391.29	80.64	17.25	< .001
Initial Performance	1256.64	147.55	8.52	< .001
Sex:Session	-852.07	129.96	-6.56	< .001
Sex:Initial Performance	-783.19	239.45	-3.27	.002
Session:Initial Performance	-884.46	115.88	-7.63	< .001
Sex:Session:Initial Performance	540.93	188.04	2.88	.006
Thrusts				
Sex	-814.82	258.37	-3.15	.003
Session	-369.76	148.93	-2.48	.016
Initial Performance	-783.16	230.37	-3.40	.001
Sex:Session	322.27	240.08	1.34	.186
Sex:Initial Performance	720.86	373.86	1.93	.059
Session:Initial Performance	284.25	214.05	1.33	.190
Sex:Session:Initial Performance	-200.99	347.38	-0.58	> .250
Clockwise Rotations				
Sex	-339.87	108.25	-3.14	.003
Session	-136.83	51.65	-2.65	.011
Initial Performance	-311.79	96.52	-3.23	.002
Sex:Session	235.54	83.25	2.83	.007
Sex:Initial Performance	334.45	156.64	2.14	.038
Session:Initial Performance	157.76	74.23	2.13	.039
Sex:Session:Initial Performance	-126.99	120.46	-1.05	> .250
Counterclockwise Rotations				
Sex	-498.44	95.40	-5.23	< .001
Session	-356.53	59.16	-6.03	< .001
Initial Performance	-495.68	85.06	-5.83	< .001
Sex:Session	271.13	95.38	2.84	.006
Sex:Initial Performance	415.39	138.05	3.01	.004
Session:Initial Performance	259.18	85.04	3.05	.003
Sex:Session:Initial Performance	-186.49	138.02	-1.35	.183

## Discussion

Traditional theories have primarily attributed sex differences in domains requiring extensive visuospatial cognition to innate biological differences between men and women; however, the present investigation found that sex differences in control performance in a video game were associated with modifiable differences in control behaviors. Consistent with previous studies, our investigation found significant sex differences in performance in an action-video game, both prior to and following extensive training. Men displayed an advantage compared to women on Control score, but women made significantly larger gains over 30 hours of training and closed the performance gap substantially. This finding was extended by our discovery that men and women also displayed differential patterns of control behaviors across training. Additionally, the behavior of initially lower-performing women differed not only from men, but also from initially higher-performing women.

Our finding that some women were controlling their ships differently from other women raises issues for the assumption that the mechanisms underlying sex differences in action-video game performance can be accounted for by sex differences in innate spatial abilities. An argument in favor of innate sex differences would have to address differences in behavior between players of the same sex with different levels of performance. Looking at the beginning of training, where sex differences were largest, we found sex differences in behavior were dependent upon whether a player was a high or low performer within his or her sex. Initially low-performing women performed notably worse than other players, and they also executed notably more behaviors per game than other players, suggesting their behaviors may have led to the significant sex differences and obscuring the fact some women were performing and behaving similarly to the men.

One possible explanation for the large sex differences could be that some women began playing SF with less efficient control behavior. For example, Towne and colleagues [45] described a strategy employed by skilled SF players in which they maneuvered their ship around the fortress in a slow clockwise orbit, maintaining a close proximity without actually crashing into the fortress. Executing this sort of plan or control strategy would require players to make very few counterclockwise rotations, as they would have to constantly rotate their ship clockwise and thrust in order to counteract the effects of centrifugal force in the frictionless game environment. The behavioral analyses revealed initially lower-performing women had a hyperactive control approach, executing more control behaviors than other players. This could be reflective of qualitatively different plans or control strategies, or perhaps more accurately the lack of a consistent plan or control strategy. However, the study providing the current data did not assess participants’ plans and strategies by collecting verbal reports on goals, so it is not possible to differentiate plans and specific strategy types among participants in the sample. Future experiments will be critical in testing the extent to which strategy selection contributes to sex differences in performance for spatially-demanding tasks like SF.

A possible alternative hypothesis is that all the participants were indeed attempting to execute a similar plan and control strategy, but individual differences in some participants’ spatial abilities rendered them unable to execute it effectively. We argue that the remarkably high number of behaviors, especially thrusts, executed by some lower-performing women makes such an argument less plausible. Some participants were executing over 1,000 ship thrusts per game, which equates to over 5 thrusts every second, on average; this is only possible if a player is continuously holding down the joystick in the forward position to keep increasing speed. In the frictionless environment of the game, this constant acceleration would be a very maladaptive gameplay style and make it difficult to gain control of the ship. An inspection of Fig 6 supports this by demonstrating that the flight path of the lower-performing woman lacks a definite path and shows essentially unpredictable flight patterns. Furthermore, this control style is most likely volitional because the joystick participants used to control the ship was spring-mounted and returned to a central neutral position if they exerted no force on it. Whereas individual differences in spatial ability may contribute to performance differences among participants using the traditional discrete ship thrusting technique, they would not account for individuals who accelerated their ship constantly. Negative transfer would seem to be a likely potential factor, with participants erroneously viewing constant acceleration as necessary for continued forward momentum, as with driving a car. Though we can only speculate on the origin of such behavior differences at this point, there appears to be some deficient understanding of the control mechanics involved, driven by qualitatively different representations of the task demands of the game. It is possible that although all the women in our sample had limited exposure to video games, perhaps some had more experience with other spatially-demanding tasks and developed spatial skills that predisposed them to selecting a control style more similar to the men, per the biopsychosocial model described by [10]. Future studies collecting detailed behavioral data and self-reported strategy information or concurrent verbal reports during gameplay could provide greater insight into the cognitive processes underlying such differences in behavior throughout training. To this end, we support the assertions of [47] and [48] that more studies examining performance involving dynamic spatial reasoning in virtual environments should look at participants’ behaviors more thoroughly rather than relying solely on performance summary information. This type of detailed behavioral data could provide better understanding of differences in the use of plans and strategies of differing effectiveness and perhaps shed some light on what specific knowledge and skills are relevant for development of superior performance in a variety of domains involving remote operation or navigation in virtual environments.

The present study has demonstrated that traditional generalizations across levels of overall performance may not fully capture the complex nature of sex differences in spatial skills and their relationship to performance in action video games. These data suggest some women were executing patterns of control behaviors that were qualitatively different from the men and other higher-performing women, casting doubt on the premise that innate differences in spatial ability primarily accounted for sex differences in control performance. Additionally, the findings show that female participants significantly changed their control behavior over time, demonstrating both the mutability of strategy with experience and the informative value of investigating participant performance at the level of frequency of individual behaviors. Much of the established literature linking spatial abilities to sex differences in domains ranging from STEM education [49] to video game play relies on accuracy or reaction time measures that provide limited insights into what men and women are actually doing and thinking while performing the tasks. Future studies of sex differences in the development of spatially-demanding complex skills should look behind summary scores and also examine differences in men and women’s behavior in order to better understand the source of these sex differences, as well as what types of training can help overcome them.

## Supporting information

S1 File Study data. Comma delimited plain text file containing data used in the present investigation.
(CSV)

S2 File Study data variable coding. Plain text file describing study data variable coding.
(TXT)
